# TRPV1 modulated NLRP3 inflammasome activation via calcium in experimental subarachnoid hemorrhage

**DOI:** 10.18632/aging.205379

**Published:** 2024-01-04

**Authors:** Keke Zhang, Zhen Qin, Jinyan Chen, Gengyin Guo, Xiaokun Jiang, Feng Wang, Jianfeng Zhuang, Zhen Zhang

**Affiliations:** 1 Department of Endocrinology, The First Affiliated Hospital of Xi'an Jiaotong University, Xi'an, Shaanxi 710061, China; 2 Department of Otolaryngology, Shandong Provincial Hospital Affiliated to Shandong First Medical University, Jinan, Shandong 250021, China; 3 Department of Clinical Laboratory, Affiliated Hospital of Shandong University of Traditional Chinese Medicine, Jinan, Shandong 250021, China; 4 Department of Neurosurgery, Shandong Provincial Hospital Affiliated to Shandong First Medical University, Jinan, Shandong 250021, China; 5 State Key Laboratory of Translational Medicine and Innovative Drug Development, Jiangsu Simcere Pharmaceutical Co., Ltd., Nanjing 210023, China; 6 Department of Neurosurgery, Qilu Hospital of Shandong University, Jinan, Shandong 250021, China

**Keywords:** subarachnoid hemorrhage, early brain injury, neuroinflammation, TRPV1, NLRP3 inflammasome

## Abstract

Neuroinflammation plays a key role in early brain injury (EBI) of subarachnoid hemorrhage (SAH), and NLRP3 inflammasome plays an important role in the development of neuroinflammation after SAH, but the mechanism of NLRP3 inflammasome activation after SAH is still unclear. TRPV1 is a non-selective calcium channel that is involved in the pathology of neuroinflammation, but its role in SAH has not been revealed. Our study showed that TRPV1 was significantly upregulated after SAH and was predominantly expressed in microglia/macrophages. Antagonism of TRPV1 was effective in ameliorating neurological impairment, brain edema, neuronal damage, and reducing the inflammatory response (evidenced by reducing the number of CD16/32 positive microglia/macrophages, inhibiting the expression of CD16, CD32, CD86, IL-1b, TNF-a and blocking NLRP3 inflammasome activation). However, this effect can be abolished by NLRP3 inflammasome antagonist MCC950. *In vitro* experiment confirmed that TRPV1 activated NLRP3 inflammasome by increasing intracellular calcium levels. In conclusion, TRPV1 mediates EBI after SAH via calcium/NLRP3, and TRPV1 is a potential therapeutic target after SAH.

## INTRODUCTION

Subarachnoid hemorrhage (SAH) is an acute cerebrovascular disease with high mortality and morbidity, mainly caused by the rupture of intracranial aneurysms [1]. Early brain injury (EBI) is one of the main factors for the poor prognosis of SAH patients. Much evidence indicates that neuroinflammation is the main pathophysiological mechanism of EBI. Inhibiting the inflammatory response after SAH can effectively alleviate EBI and significantly promote the recovery of neurological function after SAH [2]. The NLRP3 inflammasome is a multi-protein complex associated with various inflammatory diseases [3]. The NLRP3 protein consists of an amino-terminal pyrin domain (PYD), a NACHT domain, and a carboxy-terminal leucine-rich (LRR) repeat domain [4]. The PYD domain of NLRP3 binds to the PYD domain of ASC and then recruits inactive caspases-1 through the interaction of ASC with the common recruitment domain (CARD) to form the NLRP3 inflammasome. Activation of the NLRP3 inflammasome cleaves pro-caspase-1 into active caspase-1, which induces the pro-inflammatory cytokine IL-1β and IL-18 process and secretion, and causes inflammatory response.

Recent studies have been gradually revealing the effects of NLRP3 on the occurrence and development of the central nervous system. In the pathological process of Alzheimer's disease, abnormally activated NLRP3 inflammasome can lead to pathological damage of neurons and accelerate the deterioration of neural function [5]. Soares et al. have reported that in a mouse model of multiple sclerosis, pharmacological inhibition of NLRP3 activation can significantly reduce neuroinflammation and promote the improvement of motor function [6]. In addition to the above-mentioned chronic neurodegenerative diseases, studies have confirmed that NLRP3 inflammasome activated after SAH, and inhibition of NLRP3 inflammasome can alleviate EBI after SAH [7, 8]. However, the mechanism of the NLRP3 inflammasome activation is very complex, including abnormal distribution of intracellular ions (potassium, calcium, and chloride ions) [9–15], oxidized mitochondrial DNA (ox-mtDNA) [16], unstable soluble Enzymes [17] and reactive oxygen species (ROS) [18, 19]. The disruption of intracellular ion homeostasis has been considered to be one of the most important factors in inducing NLRP3 inflammasome activation. Recent studies have shown that calcium ions influx or migration plays an important role in the activation of the NLRP3 inflammasome [20–22], however, whether calcium ions are involved in NLRP3 inflammasome activation after SAH and the underlying mechanisms involved remains unclear.

TRPV1, also known as vanilloid receptor type 1 (VR1), is a member of the transient receptor potential channel protein family. Previous studies have focused on the role of TRPV1 in sensory transmission from nociceptive neurons in the peripheral nervous system [23]. Recent reports indicated that TRPV1 was widely distributed in the central nervous system, and it may involve regulating the neuroinflammatory processes [24]. Moreover, some studies have pointed out that TRPV1 regulated neuroinflammation by activating the NLRP3 inflammasome [25–27]. Herein, we speculate that TRPV1 and associated NLRP3 inflammasome might play an essential role in SAH-induced neuroinflammation.

## MATERIALS AND METHODS

### Mouse models of SAH and drugs administration

C57BL/6 mice (male, 22–25 g) were purchased from Vital River Laboratory Animal Technology (Beijing, China). The procedures involved in mice were conformed to the guidelines of the National Institutes of Health on the care and use of laboratory animals and approved by the Institutional Animal Care and Use Committee of Shandong First Medical University.

SAH model was constructed via endovascular perforation as the previous study [28]. Briefly, the mice were anesthetized using 5% isoflurane, and then the anesthesia was maintained at 2% isoflurane during the entirety of the experiment, the nylon suture was passed through the external carotid artery to the bifurcation of the anterior and middle artery and ultimately punctured to cause blood to flow into the subarachnoid space.

Mice were injected with 30 mg/kg capsazepine (CPZ, MedChemExpress, Cat#HY-15640) subcutaneously or 10 mg/kg capsaicin (CAP, MedChemExpress, Cat# HY-10448) intraperitoneally post-modeling to inhibit or active TRPV1 [26, 29], respectively. MCC950 (MedChemExpress, Cat# HY-12815) (40 mg/kg) were intraperitoneally injected post-modeling and 12 h later [8]. The drugs above were all purchased from MedChemExpress (NJ, USA).

### Magnetic-activated cell sorting (MACS) and qPCR analysis

Ipsilateral hemispheres were harvested at 24 h post-SAH, dissociated mechanically using a glass homogenizer, and filtered into a 70-μm strainer to obtain a single-cell suspension. The single-cell suspension was isolated with 30% Percoll (Cytiva, Cat#17089101) solutions, incubated with CD11b microbeads (Miltenyi Biotec, Germany, 130-126-725), and then divided into CD11b positive and negative cells.

Total mRNA of tissues and cells was extracted using TRIzol^™^ Reagent (Life Technologies, CA, USA), used to synthesize cDNA using Evo M-MLV Reverse Transcriptase (Takara, Japan, RR420A), and then performed the qPCR analysis using SYBR Premix Ex Taq^™^ Kit (Takara, Japan, RR036A) according to manufacturer’s protocol. The primers were listed in Supplementary Table 1.

### Brain water content

Twenty-four hours post-operation, the mice were euthanized, and the left hemisphere were promptly excised and immediately weighed to determine its wet weight (WW). Subsequently, the left hemisphere was dehydrated in a dry bath at 105°C for 72 hours to ascertain their dry weight (DW). The water content for each brain segment was calculated using the formula: ((WW − DW)/WW) × 100%.

### Neurobehavioral assessment

Neurobehavioral assessment containing modified Garcia and beam balance test was performed 24 h post-SAH to evaluate neurological function [30]. Modified Garcia and beam balance scores ranged from 3 to 18 and 0 to 4 respectively, with higher scores indicating better neurological function.

### Histological staining analysis

Mice were anesthetized and perfused sufficiently with 4% paraformaldehyde (PFA) at 24 h after SAH to harvest the brain tissues. The brain tissues were fixed in 4% PFA for 24 h, dehydrated in 30% sucrose solution, embedded in OCT compound, and then sliced into 10 μm coronal sections for immunofluorescence and Nissl staining analysis.

For immunofluorescence staining analysis, mice brain sections were blocked and permeabilized with 5% bovine serum containing 0.3% Triton-X100 for 1 h at room temperature and incubated with primary antibodies overnight at 4°C followed by 1 hour incubation at room temperature with the corresponding secondary antibody. The primary antibodies included anti-Iba-1 (Abcam, Cambridge, UK, ab-5076), anti-TRPV1 (Abcam, Cambridge, UK, ab203103), and anti-CD16/CD32 antibody (Biolegend, CA, USA, 101329).

For Nissl staining, mice brain sections were stained with 0.5% cresyl violet for 0.5 h at room temperature and dehydrated with ethanol absolute [31]. The images were analyzed using Image J (NIH).

### Western blot

Proteins of tissues and cells were extracted using RIPA lysis buffer (Beyotime, Shanghai, China, P0013B), separated by SDS-PAGE, transferred into PVDF membrane, blocked with 5% non-fat powdered milk, incubated with primary antibodies overnight at 4°C followed by 1 hour incubation at room temperature with the corresponding secondary antibody. Then, the PVDF membrane was visualized using the ECL Plus chemiluminescence reagent kit. The primary antibodies included anti-β-actin (Proteintech, Hubei, China, 60008-1-Ig), anti-ZO-1 (Santa Cruz, TX, USA, sc-33725), anti-claudin-5 (Santa Cruz, TX, USA, sc-28670), anti-occludin (Santa Cruz, TX, USA, sc-133256), anti-NLRP3(Abcam, Cambridge, UK, ab210491), anti-ASC (Santa Cruz, TX, USA, sc-271054), anti-caspase-1 p20 (Santa Cruz, TX, USA,sc-22165). The protein expression was quantified using Image J (NIH).

### ELISA

The total protein content of tissues and cell culture supernatants was determined via BCA assay (Beyotime, China). The expression level of IL-1β was detected using ELISA kit (Boster, Wuhan, China, EK0394) according to the manufacturer’s protocol.

### *In vitro* SAH model

Murine BV2 cells were stimulated by 200 μM hemin (Sigma-Aldrich, MO, USA, H9039) to establish the SAH model *in vitro*. Meanwhile, BV2 cells were treated with 10 μM CAP [32] and BAPTA-AM [26] to investigate the mechanism of the TRPV1-activated NLRP3 inflammasome.

### Calcium concentration detection

Calcium concentration was detected as previously reported [33]. Briefly, BV2 cells were incubated with 4 μM Fura-2/AM (YEASEN, Shanghai, China, 40702ES50) for 30min, and captured the fluorescence intensities when excited at 340 and 380 nm and emitted at 510 nm. The calcium concentration was calculated as followed: the fluorescence intensities at 340 nm/ the fluorescence intensities at 380 nm.

### Statistical analysis

The data were analyzed by SPSS 22.0 and GraphPad Prism 8.0 to test whether met the normal distribution and variance homogeneity by Shapiro-Wilk and Levene methods, respectively. When met, data were analyzed by Student’s *t*-test (two groups) or one-way analysis of variance (ANOVA) with Tukey’s post hoc contrasts (more than three groups); otherwise, data were analyzed by Mann–Whitney nonparametric test (two groups) and Kruskal-Wallis test with post hoc contrast by the Dunn-Bonferroni test (more than three groups).

### Availability of data and materials

The datasets used during the present study are available from the corresponding author upon reasonable request.

## RESULTS

### TRPV1 upregulated post-SAH

**Figure 1:**
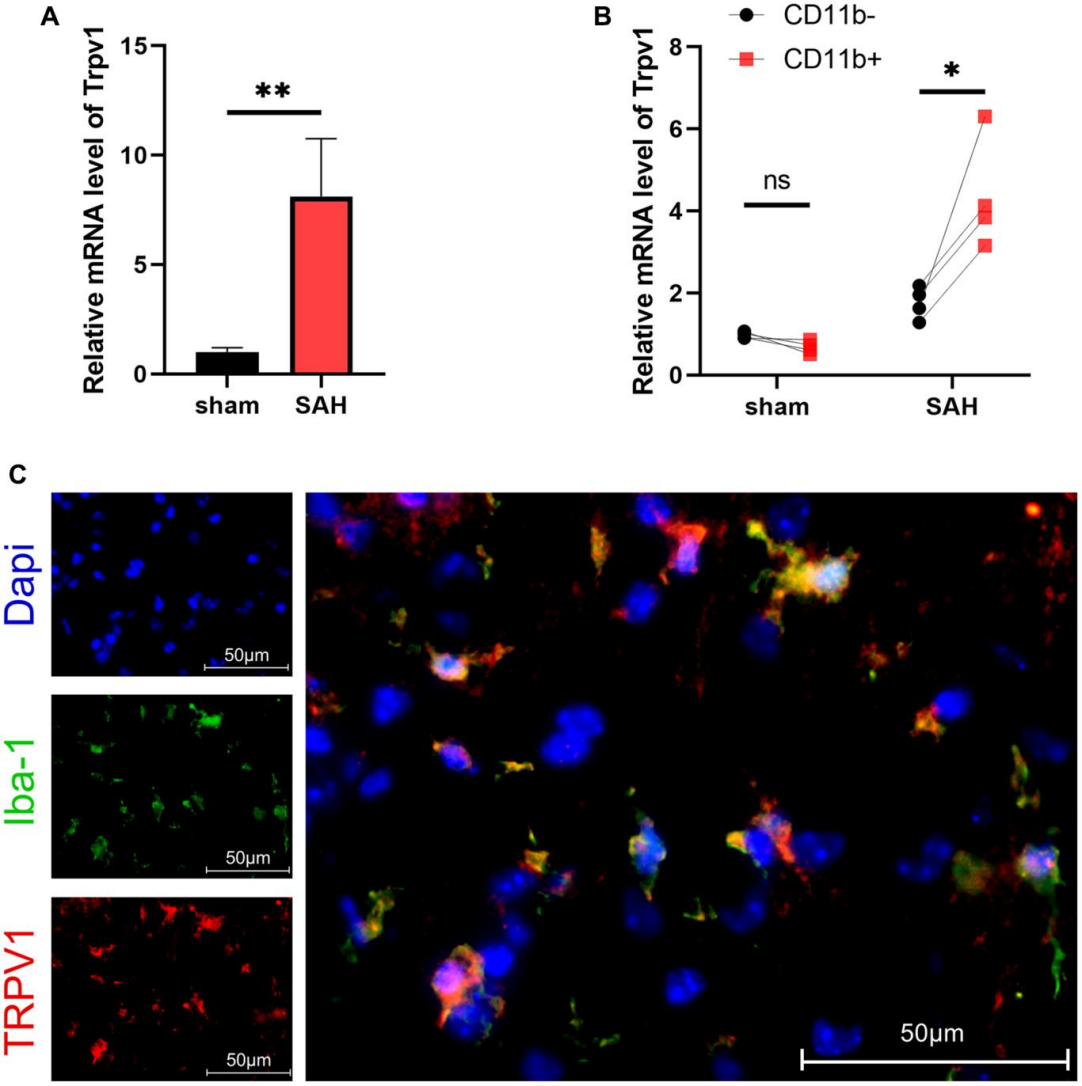
**The expression and distribution of TRPV1.** (**A**, **B**) The mRNA level of Trpv1 in brain tissues or CD11b positive/negative cells isolated via MACS was assessed by qPCR at 24 h post-SAH. (**C**) Representative immunofluorescence images showed the co-localization of TRPV1 with Iba-1. Abbreviation: Abbreviation: no significance; ^*^p < 0.05 and ^**^p < 0.01. Scale bar = 50 μm.

TRPV1 plays crucial roles in various physiological and pathological processes, including sensation of mechanical stimuli, voltage sensitivity, response to chemical substances, immune activation, modulation of temperature, gastrointestinal function regulation, and involvement in tumorigenesis. The results of qPCR showed that the mRNA level of Trpv1 was increased at 24 hours after SAH (Figure 1A). Using MACS to separate the brain cells into CD11b positive and negative cells, it was found that Trpv1 changed most significantly in the CD11b positive cells (Figure 1B). Consistently, immunofluorescence staining demonstrated that TRPV1 was mainly expressed in microglia/macrophages (Figure 1C).The expression status of TRPV1 in microglia and macrophages deserves further study.

### Antagonism of TRPV1 attenuated neurological dysfunction

**Figure 2:**
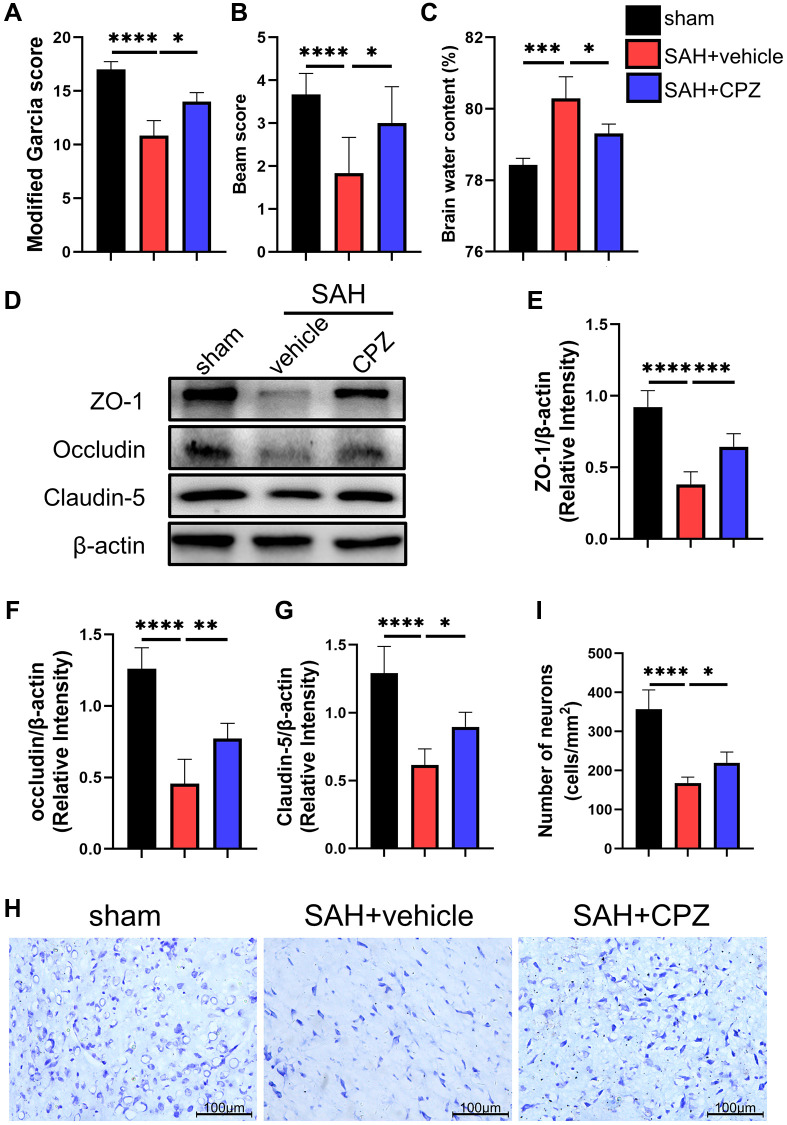
**Effect of TRPV1 on neurological function, brain edema, and neuron injury.** (**A**, **B**) Neurological function at 24 h post-SAH was evaluated via modified Garcia and beam score. (**C**) The water content of the left hemisphere at 24 h post-SAH. (**D**–**G**) The relative protein expression of ZO-1, Occludin, and Claudin-5 at 24 h post-SAH was assessed via western blot. (**H**, **I**) The neuron number at 24 h post-SAH was assessed via Nissl stain. ^*^p < 0.05, ^**^p < 0.01, ^***^p < 0.001 and ^****^p < 0.0001. Scale bar = 100 μm.

We conducted follow-up studies to investigate the effect of TRPV1 on neurological impairment, CPZ, a selective TRPV1 antagonist, was introduced. Neurobehavioral scores including modified Garcia scores and beam scores showed severe neurological dysfunction after SAH when compared with the sham group. However, blockage of TRPV1 with CPZ improved the neurological dysfunction (Figure 2A, 2B). The administration of CPZ dramatically reduced the brain water content and upregulated the expression of tight junction proteins including ZO-1, occluding, and claudin-5 (Figure 2C–2G). Nissl staining also showed that CPZ increased the number of neurons (Figure 2H, 2I).

### Antagonism of TRPV1 alleviated the neuroinflammation

**Figure 3:**
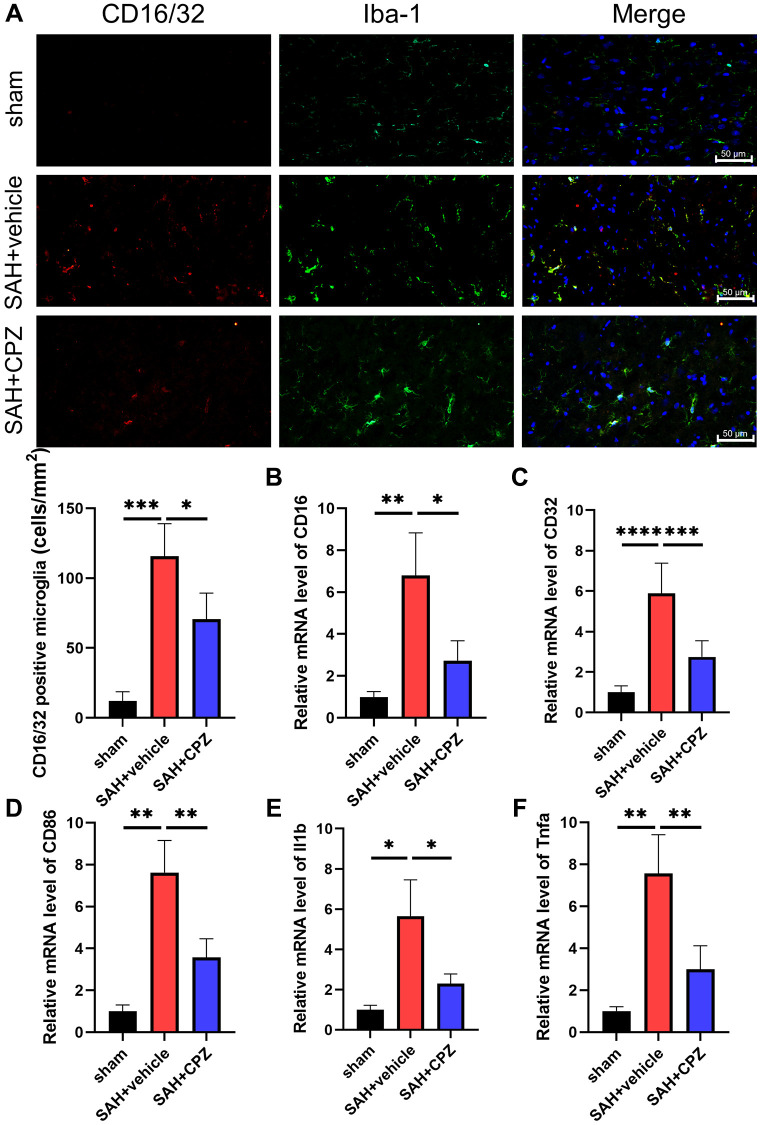
**Effect of TRPV1 on inflammation response after SAH.** (**A**) The immunofluorescence showed the number of CD16/32 positive microglia/macrophages at 24 h post-SAH. (**B**–**F**) The mRNA level of CD16, CD32, CD86, IL-1β, and TNF-α in brain tissues at 24 h post-SAH. ^*^p < 0.05, ^**^p < 0.01, ^***^p < 0.001 and ^****^p < 0.0001. Scale bar = 50 μm.

**Figure 4:**
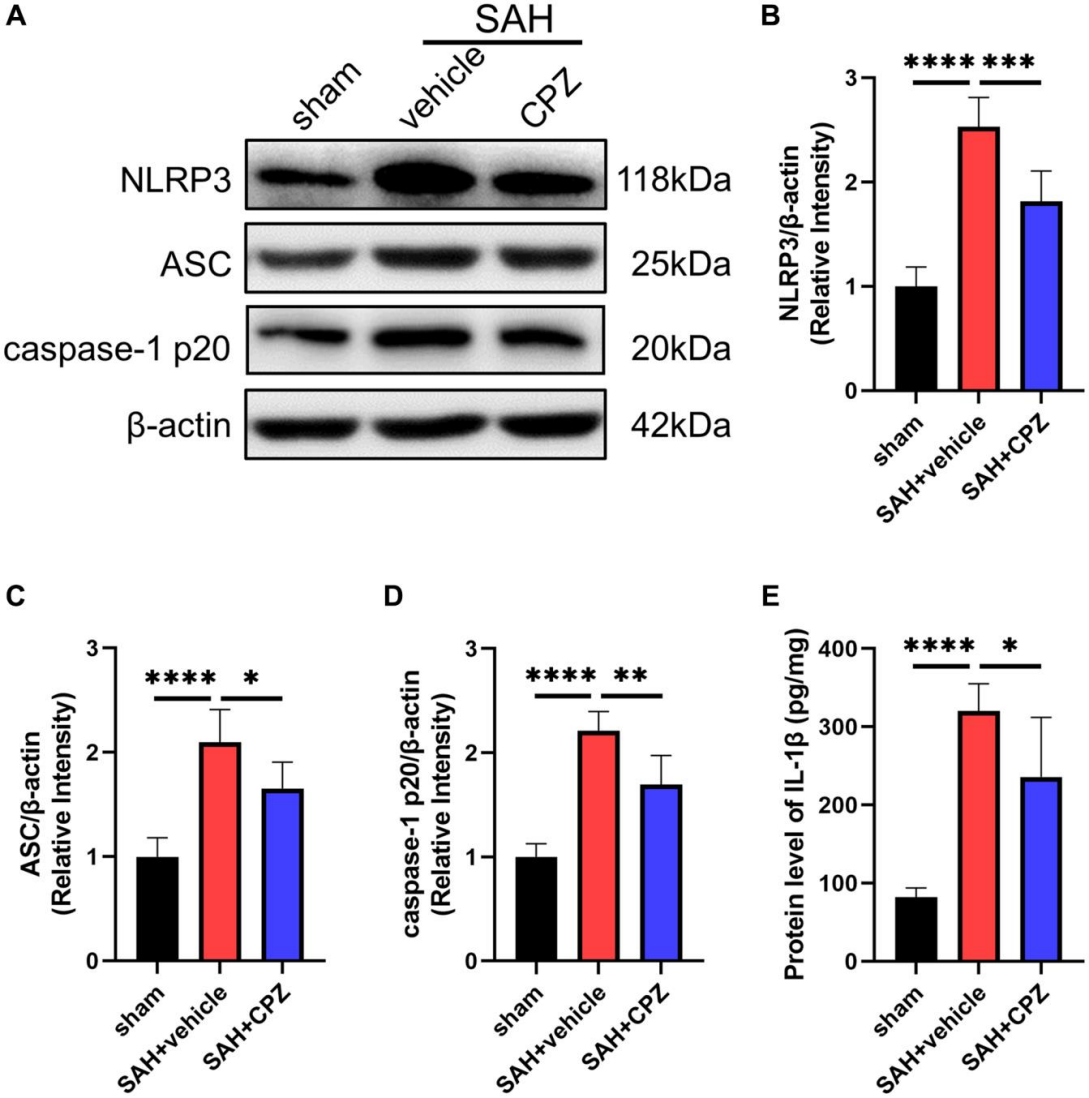
**Effect of TRPV1 on NLRP3 inflammasome activation.** (**A**–**D**) The relative protein expression of NLRP3, ASC, and caspase-1 p20 at 24 h post-SAH was assessed via western blot. (**E**) The protein level of IL-1β at 24 h post-SAH was assessed via ELISA. ^*^p < 0.05, ^**^p < 0.01, ^***^p < 0.001 and ^****^p < 0.0001.

We have learned that TRPV1 activation affects nerve function damage. Can the inhibition of TRPV1 alleviate nerve function damage? We have conducted further research on the possible mechanism of TRPV1 in alleviating nerve function damage. Immunofluorescence staining demonstrated that the number of CD16/32 positive microglia/macrophages increased after SAH and reduced after treatment with CPZ (Figure 3A). Consistent with immunofluorescence staining, the mRNA level of CD16, CD32, CD86, IL-1β, and TNF-α was elevated in the SAH+vehicle group, but was reduced in the SAH+CPZ group (Figure 3B–3F). Subsequently, we investigated whether TRPV1 was involved in the activation of the NLRP3 inflammasome. The western blot results showed that the expression of NLRP3, ASC, and caspase-1 p20 were increased in the SAH+vehicle group and reduced in the SAH+CPZ group (Figure 4A–4D). The level of IL-1β showed a similar change as determined via ELISA (Figure 4E).

### TRPV1 enhanced neuroinflammation via the NLRP3 inflammasome

**Figure 5:**
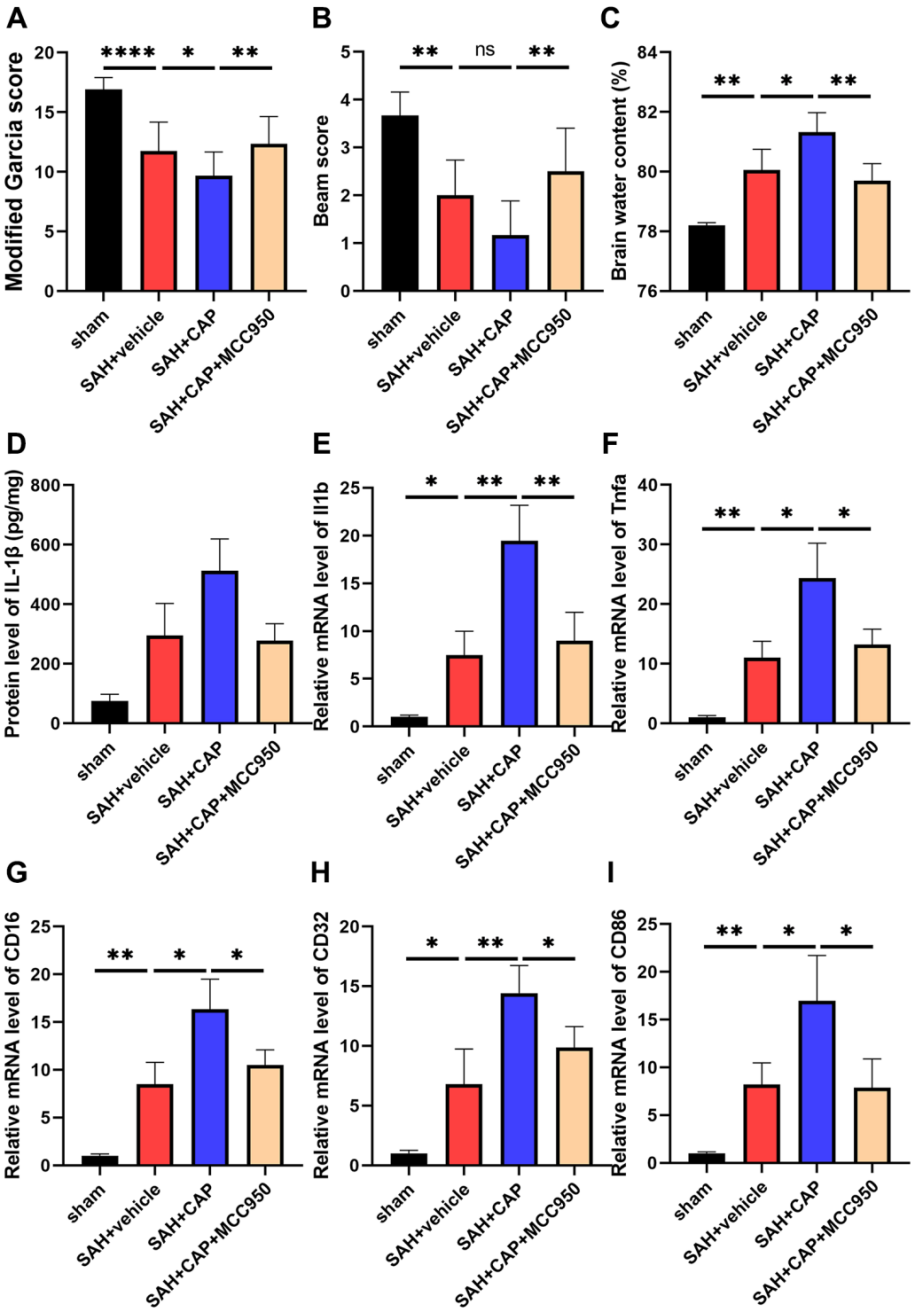
**Effect of NLRP3 on TRPV1 mediated neurological dysfunction and inflammatory response at 24 h post SAH.** (**A**, **B**) Neurological function was valued via modified Garcia and beam score. (**C**) The water content of the left hemisphere. (**D**) The protein level of IL-1β was assessed via ELISA. (**E**–**I**) The mRNA level of CD16, CD32, CD86, IL-1β, and TNF-α in brain tissues was assessed via qPCR. Abbreviation: no significance; ^*^p < 0.05, ^**^p < 0.01 and ^****^p < 0.0001.

To further investigate the effect of NLRP3 inflammasome on TRPV1-induced neuroinflammation, CAP, a selective TRPV1 agonist, and MCC950, a selective TRPV1 antagonist, were used to activate TRPV1 and inhibit NLRP3, respectively. Administration of CAP decreased the modified Garcia and beam scores and increased the brain water content compared with the SAH+vehicle group, while inhibition of NLRP3 with MCC950 reversed the aggravation of neurobehavioral assessment and brain water content (Figure 5A–5C). Data from ELISA and qPCR confirmed that MCC950 inhibited the elevation of the level of IL-1β, TNF-α, CD16, CD32, and CD86 induced by CAP (Figure 5D–5I).

### TRPV1 activated NLRP3 inflammasome via calcium

**Figure 6:**
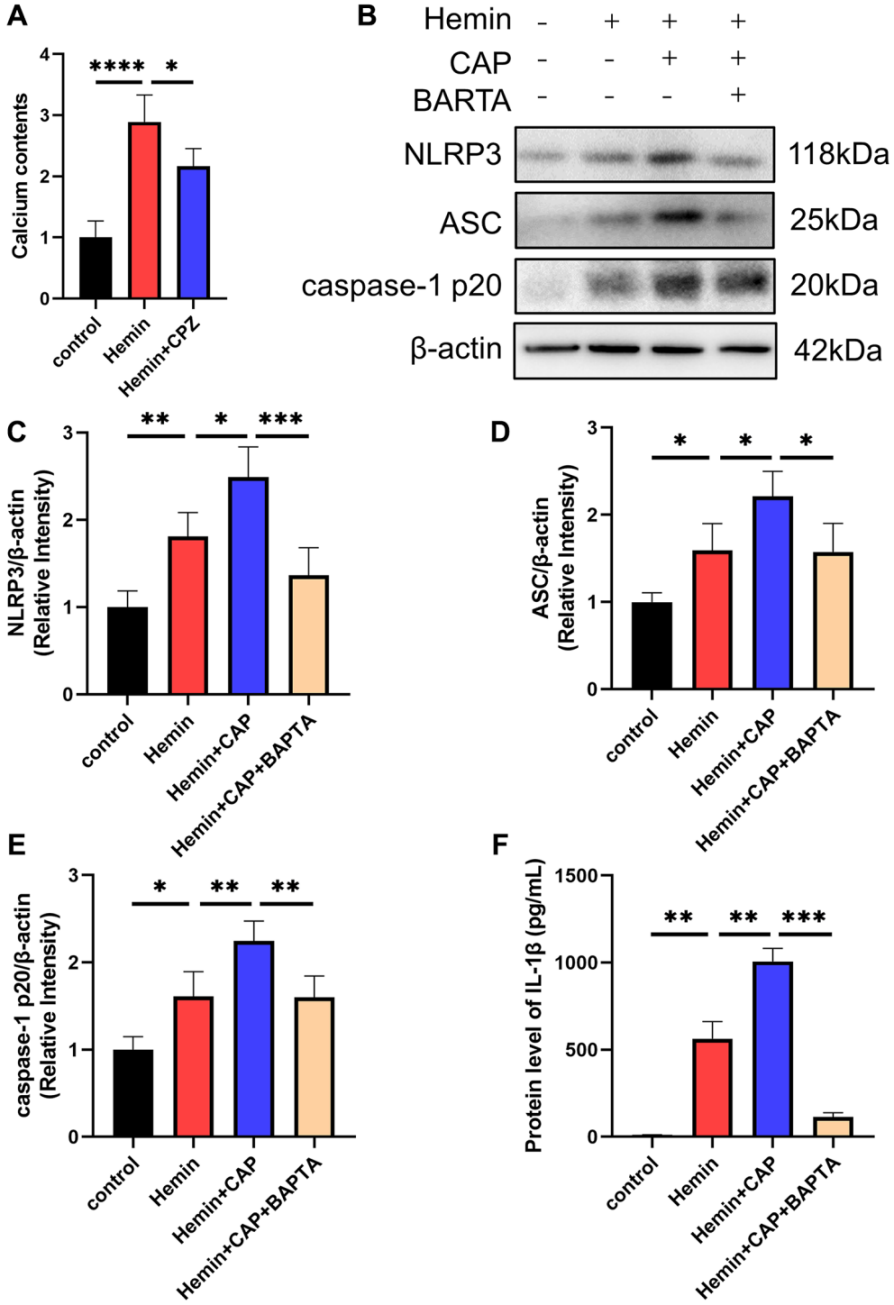
**Effect of calcium on TRPV1 mediated NLRP3 inflammasome activation.** (**A**) The calcium content of BV2 cells was assessed at 24 h after modeling. (**B**–**E**) The relative protein expression of NLRP3, ASC, and caspase-1 p20 in BV2 cells at 24 h post modeling was assessed via western blot. (**F**) The protein level of IL-1β in BV2 cells supernatant at 24 h post modeling was assessed via ELISA. ^*^p < 0.05, ^**^p < 0.01, ^***^p < 0.001 and ^****^p < 0.0001.

Given TRPV1’s essential role in regulating calcium homeostasis and the involvement of calcium in NLRP3 inflammasome activation, calcium was introduced to investigate the underlying mechanism by which TRPV1 induced NLRP3 inflammasome activation. The calcium content of microglia was dramatically increased when stimulated with hemin, while it was reduced when treated with CPZ (Figure 6A). Chelation of calcium via BAPTA-AM reversed the increased levels of NLRP3, ASC, caspase-1 p20, and IL-1β induced by hemin (Figure 6B–6F).

## DISCUSSION

As TRPV1 is not only expressed in peripheral sensory neurons but also widely expressed in the central nervous system, attention has gradually moved beyond its classical role as a pain sensor to focus on its role in the central nervous system [34–36]. Although many studies have pointed to the indispensability of TRPV1 in the central nervous system, particularly in the pathology of the inflammatory response, it is a puzzle to conclude exactly what role it plays, as it exhibits deteriorated or protective effects in the context of diseases [37, 38]. For deteriorated effects, the selective TRPV1 antagonists CPZ inhibited the morphine-induced increased expression of p38 MAPK and NF-κB [39]. Additionally, Miyanohara et al. confirmed that both TRPV1 knockout and CPZ showed an alleviated neurological function after stroke [40]. For the protective effects, TRPV1 suppressed the oxidative stress and enhanced the endogenous production of a neurotrophic factor in the neurodegeneration diseases including Parkinson’s disease [41], vascular dementia [42], and Huntington’s disease [43]. The mechanism was at least in part related to the anti-inflammatory effect [44–46]. Hence, it is essential to determine the specific effect of TRPV1 in the context of SAH. We found that CPZ inhibited TRPV1 alleviated the neurological function injury, BBB disruption and reduced the pro-inflammatory microglia, and was associated with the inflammatory response, indicating the deteriorated effects of TRPV1 after SAH.

Since TRPV1 was mainly expressed in microglia/macrophages and microglia/macrophages were thought to be one of the most important factors mediating the inflammatory response after SAH, we hypothesize that the brain injury effect of TRPV1 after SAH might be mainly due to its pro-inflammatory effect. The pro-inflammatory mechanism of TRPV1 remains unclear, however, there were some recent studies linking it to NLRP3 inflammasome [25–27, 47]. Moreover, growing evidence suggested that the NLRP3 inflammasome was involved in the inflammatory response after SAH, that inhibition of the NLRP3 inflammasome significantly alleviates neurological dysfunction, and that the NLRP3 inflammasome activation mechanisms after SAH related to SIRT1, NRF2, AMPK, etc., [8, 48–50]. However, the effect of TRPV1 on the NLRP3 inflammasome activation has not been determined in SAH. Our data confirmed that the expression of NLRP3 inflammasome-related proteins NLRP3, ASC, and caspase-1 p20 were down-regulated after pharmacological antagonism of TRPV1, and the level of inflammatory cytokine IL-1β was significantly decreased. Furthermore, inhibiting the NLRP3 inflammasome abolished the TRPV1 mediating the neurological dysfunction and inflammatory response. Since calcium ions can mediate NLRP3 inflammasome activation and TRPV1 can act as a non-selective calcium channel, we next explored the role of calcium ions on TRPV1-mediated NLRP3 inflammasome activation *in vitro*. The data showed that suppressing the TRPV1 reduced the calcium ion contents in BV2 cells. Moreover, chelated calcium ions reversed the TRPV1 mediating NLRP3 inflammasome activation. Previous studies have found that two mechanisms might be involved in calcium-mediated NLRP3 inflammasome activation: (1) promoting the interaction between NLRP3 and ASC to mediate the activation of NLRP3 inflammasome; (2) causing mitochondrial calcium overload that leads to mitochondrial damage, which in turn released ROS, ox-mtDNA to activate the NLRP3 inflammasome [51], but the mechanism under TRPV1-mediated calcium influx and NLRP3 activation remain to be defined. Zhang et al. revealed that calcium ion influx via TRPV1 directly bound to PP2A thus to active NLRP3 inflammasome in EAE [26], however, the specific mechanism in SAH needs further study.

**Figure 7:**
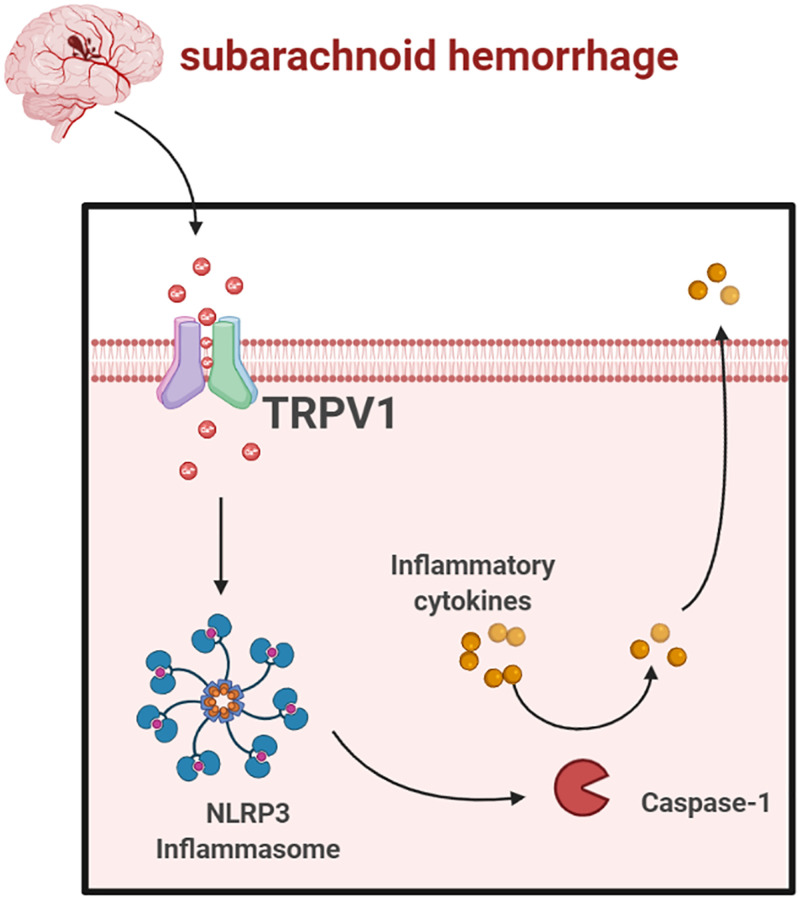
Schematic mechanism of TRPV1 activated NLRP3 inflammasome post-SAH (https://www.biorender.com/).

In conclusion, our data showed that TRPV1 modulated NLRP3 inflammasome activation via calcium and that antagonizing TRPV1 significantly improves EBI after SAH (Figure 7). Targeting TRPV1 might be a promising therapeutic strategy for SAH.

## Supplementary Materials

Supplementary Table 1
