# Supplementary material for: TRPV1 modulated NLRP3 inflammasome activation via calcium in experimental subarachnoid hemorrhage
Source: Aging (Albany NY). 2024 Jan 4;16(2):1096–110. doi: 10.18632/aging.205379 (PMC10866436; doi:10.18632/aging.205379)
Supplement: Supplementary Table 1 [file aging-16-205379-s001.pdf]

SUPPLEMENTARY TABLE

Supplementary Table 1. Sequence of primers for qPCR.

Gene		Primer sequence
β-actin	F	5′-AGGCATTGTGATGGACTCCG-3′
	R	5′-AGCTCAGTAACAGTCCGCCTA-3′
TRPV1	F	5′-CGAGGATGGGAAGAATAACTCACTG-3′
	R	5′-GGATGATGAAGACAGCCTTGAAGTC-3′
IL-1β	F	5′-GCCACCTTTTGACAGTGATGAG-3′
	R	5′-GGAGCCTGTAGTGCAGTTGT-3′
CD86	F	5′-TTACGGAAGCACCCACGATG-3′
	R	5′- TGTCAGCGTTACTATCCCGC -3′
CD16	F	5′-ACTGTGGTTGGCTTTTGGGAT-3′
	R	5′-GAGTGATTTCTGACTGGCTGCTG-3′
CD32	F	5′-CCAGAAAGGCCAGGATCTAGTG-3′
	R	5′-GGGAACCAATCTCGTAGTGTCTGT-3′
TNFα	F	5′-ATCGGTCCCAACAAGGAGGA-3′
	R	5′- CGCTTGGTGGTTTGCTACG-3′
